# Fusion of Wild-Type Mesoangioblasts with Myotubes of mtDNA Mutation Carriers Leads to a Proportional Reduction in mtDNA Mutation Load

**DOI:** 10.3390/ijms24032679

**Published:** 2023-01-31

**Authors:** Ruby Zelissen, Somaieh Ahmadian, Joaquin Montilla-Rojo, Erika Timmer, Monique Ummelen, Anton Hopman, Hubert Smeets, Florence van Tienen

**Affiliations:** 1 Department of Toxicogenomics, Maastricht University Medical Centre+, 6229 HX Maastricht, The Netherlands; 2 School for Developmental Biology and Oncology (GROW), Maastricht University Medical Centre+, 6229 HX Maastricht, The Netherlands; 3 Department of Molecular Cell Biology, Maastricht University Medical Centre+, 6229 HX Maastricht, The Netherlands; 4 Anatomy and Physiology, Department Clinical Sciences, Faculty of Veterinary Medicine, Utrecht University, 3584 CS Utrecht, The Netherlands; 5 School for Mental Health and Neurosciences (MHeNS), Maastricht University Medical Centre+, 6229 HX Maastricht, The Netherlands

**Keywords:** mtDNA disease, mtDNA mutation, myogenic stem cell therapy, myotube fusion, mesoangioblast

## Abstract

In 25% of patients with mitochondrial myopathies, pathogenic mitochondrial DNA (mtDNA) mutation are the cause. For heteroplasmic mtDNA mutations, symptoms manifest when the mutation load exceeds a tissue-specific threshold. Therefore, lowering the mutation load is expected to ameliorate disease manifestations. This can be achieved by fusing wild-type mesoangioblasts with mtDNA mutant myotubes. We have tested this in vitro for female carriers of the m.3271T>C or m.3291T>C mutation (mutation load >90%) using wild-type male mesoangioblasts. Individual fused myotubes were collected by a newly-developed laser capture microdissection (LCM) protocol, visualized by immunostaining using an anti-myosin antibody. Fusion rates were determined based on male-female nuclei ratios by fluorescently labelling the Y-chromosome. Using combined ‘wet’ and ‘air dried’ LCM imaging improved fluorescence imaging quality and cell yield. Wild-type mesoangioblasts fused in different ratios with myotubes containing either the m.3271T>C or the m.3291T>C mutation. This resulted in the reduction of the mtDNA mutation load proportional to the number of fused wild-type mesoangioblasts for both mtDNA mutations. The proportional reduction in mtDNA mutation load in vitro after fusion is promising in the context of muscle stem cell therapy for mtDNA mutation carriers in vivo, in which we propose the same strategy using autologous wild-type mesoangioblasts.

## 1. Introduction

The oxidative phosphorylation (OXPHOS) system in the mitochondrial inner membrane generates the bulk of cellular energy in the form of ATP. The OXPHOS system is under the dual genetic control of the nuclear genome and the mitochondrial DNA (mtDNA), and mutations in either genome can cause mitochondrial dysfunction, primarily affecting ATP production, which can result in mitochondrial myopathy (MM). In general, 1:5000 people are affected by mitochondrial myopathy, which has a strong negative effect on their quality of life and for which no therapy is currently available [1]. In 25% of the cases, MM is caused by a pathogenic variant in the mtDNA. The pathogenic variant is generally heteroplasmic, meaning the co-existence of both mutant and wild-type mtDNA copies in a cell. Although a negative correlation is observed between the mtDNA mutation load and OXPHOS functioning, clinical symptoms manifest when the mutation load exceeds a tissue-specific threshold [2]. Despite a high mtDNA mutation load in muscle fibres, myogenic stem cells, such as mesoangioblasts (MABs) and satellite cells in carriers of sporadic or inherited mtDNA mutations, have been demonstrated to display no or a very low (<10%) mtDNA mutation load in some (point mutations) or all (deletion) carriers [3,4]. In line with this, Spendiff et al. demonstrated that satellite cells from patients with large single mtDNA deletions gradually lost the deletion upon differentiation towards myoblasts [5]. Moreover, Shoubridge et al. demonstrated the presence of mutation-free resident satellite cells in a patient with Kearns–Sayre syndrome due to the pathogenic variant m.12315G>A. This variant was absent in the newly formed neural cell adhesion molecule positive (NCAM+) myofibers at a site of induced muscle regeneration after biopsy [6].

While satellite cells and myoblasts may be applicable for muscle regeneration upon local intra-muscular administration, MABs are suitable for systemic myogenic stem cell therapy because of their ability to cross the blood vessel wall. Intra-arterial delivery of donor MABs transplantation has already been demonstrated to be effective in a Duchenne Muscular Dystrophy dog model [7] and relatively safe in phase I/II trials in DMD patients [8]. A phase I clinical trial is currently ongoing in which autologous MABs displaying a low m.3243A>G mutation load are intra-arterially delivered in m.3243A>G mutation carriers [9]. Our hypothesis is that fusion of wild-type, mtDNA mutation-free MABs with myotubes containing a high percentage of mutated mtDNA is expected to lower the mtDNA mutation load in those fibres, preferably below the threshold, and improve mitochondrial function. Previously, Boulet et al. demonstrated in vitro that COX protein levels and enzyme activity were restored to normal after mixing 15% wild-type myoblasts with homoplasmic m.8344A>G mutant myoblast clones [10]. However, in this study, the total cell culture was isolated and analysed. Detailed analysis of the effect on the mtDNA mutation load upon fusion between mutant and wild-type myoblasts at single myotube level was not possible with this setup. In the present study, we aim to demonstrate that wild-type human MABs are able to fuse in vitro with human myotubes containing a high mtDNA mutation load. We will assess the mtDNA mutation load in individual myotubes using a new protocol for imaging and laser capture microdissection of myosin-stained myotubes. 

## 2. Results

### 2.1. Myotube-MABs Fusion Events Can Be Visualised by Using a Y-Chromosome FISH Probe

**Figure 1:**
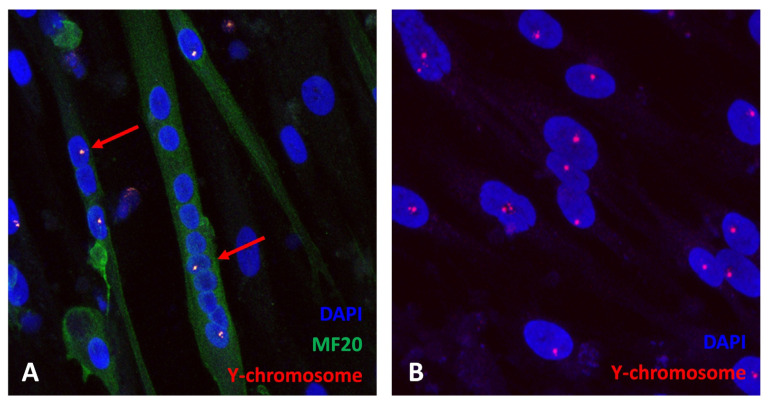
FISH Y-chromosome probe (red), MF20 staining (green), DAPI (blue). (**A**) Male mesoangioblast fusion with a female mesoangioblast and a female myotube (red arrow), (**B**) Y-chromosome FISH staining efficiency.

To quantify the fusion events between mutant myotubes and wild-type MABs, we mixed cell cultures of the opposite gender, namely female mutant MABs and myotubes (6 days after induction of myogenic differentiation) and male wild-type MABs. This enabled visualisation and quantification of the fusion events based on the number of Y-chromosome positive and Y-chromosome negative nuclei in a myotube after fluorescent in situ hybridisation (FISH) of a Y-chromosome probe in combination with immunostaining of a myotube marker (MF20; Figure 1A). To exclude the possibility of analysing false-negative male nuclei, we validated the Y-chromosome FISH staining in a pure population of 107 male MABs, which showed FISH Y-chromosome signal in all 107 nuclei, indicating maximal staining efficiency (Figure 1B).

### 2.2. Mutation-Free MABs Fuse Efficiently In Vitro with MABs and Myotubes Carrying mtDNA Mutations

**Figure 2:**
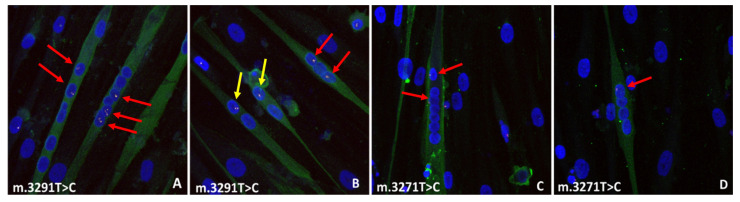
Fusion events between control MABs and MUT1 or MUT2 mutant myotubes. (**A**,**B**) control MABs fusion with m.3291T>C myotubes (red arrows), and control MABs fusion with MUT2 (m.3291T>C) MABs (yellow arrows) (**C**,**D**) control MABs fusion with MUT1 (m.3271T>C) myotubes (red arrows).

After verifying that fusion events took place between female mutant myotubes (Y-chromosome negative) and male (Y-chromosome positive) wild-type MABs, we analysed the fusion capacity in more detail. To this end, mutant MABs with either 94% m.3271T>C mutation load (MUT1) or 92% m.3291T>C mutation load (MUT2) were differentiated into myotubes. After 6 days of myogenic differentiation, male wild-type MABs were added, and the mixed culture was further differentiated until fixation at day 11. Myotubes were identified by MF20 staining, and Y-chromosome FISH staining was used to identify nuclei derived from the male wild-type MABs. As shown in Figure 2, the wild-type MABs were able to fuse in vitro with both m.3271T>C (MUT1) and m.3291T>C (MUT2) mutant myotubes containing ≥2 female nuclei (Figure 2A,C,D, red arrows). In addition, fusion and myogenic differentiation of a wild-type MAB and a mutant MAB was observed, resulting in MF20-positive myotubes consisting of one male (wild-type) and one female (MUT1 or MUT2) nucleus (Figure 2B, yellow arrows). 

**Figure 3:**
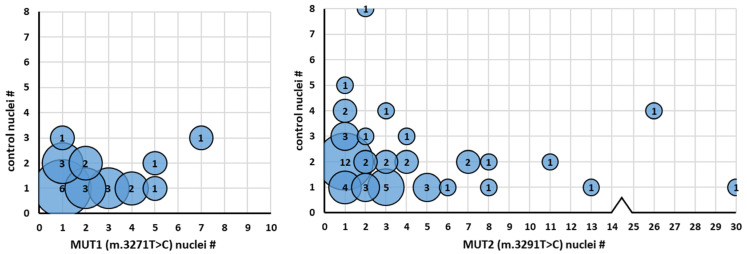
Individual fusion ratios of control MABs with m.3271T>C and m.3291T>C MABs and myotubes. X-axis: number of mutant nuclei per myotube, Y-axis: number of control nuclei per myotube. The number of observations is shown in circles. MUT1: *n* = 23, MUT2: *n* = 52.

Table 1 shows the distribution of nuclei in wild-type and mutant MABs, and wild-type, mutant and fused myotubes within the mixed cultures at differentiation day 11. We observed a presence of 13.7% Y-chromosome positive nuclei in the MUT1 + wild-type mixed culture and 26.7% in the MUT2 + wild-type mixed culture. Of the Y-chromosome positive nuclei, respectively, 30.6% and 48.3% are present in either purely WILD-TYPE or WILD-TYPE + MUT fused myotubes, demonstrating that administrated wild-type MABs are able to adhere and differentiate into differentiating myotubes in an in vitro myogenic environment. Of all fused myotubes, respectively 56.6% and 57.7% were formed by the fusion of wild-type MABs with (nascent) MUT1 or MUT2 myotubes, as defined by the fusion of ≥1 wild-type nuclei with ≥2 mutant nuclei. The remaining 43.5% and 42.3% were formed by wild-type MABs- MUT MABs fusion, defined by the presence of ≥1 wild-type nuclei with 1 mutant nucleus. To study the distribution of the fusion events in more detail, we determined the number of wild-type Y-chromosome-positive nuclei per fused myotube (Figure 3). The average wild-type to mutant ratio for wild-type/MUT1 myotubes was 42.0% ± 17.1% (mean ± stdev) and 45.4% ± 23.4% (mean ± stdev) for wild-type/MUT2.

### 2.3. Individual Myotubes Can Be Isolated for mtDNA Mutation Load Assessment with Fluorescent Laser-Capture Microdissection (LCM)

**Figure 4:**
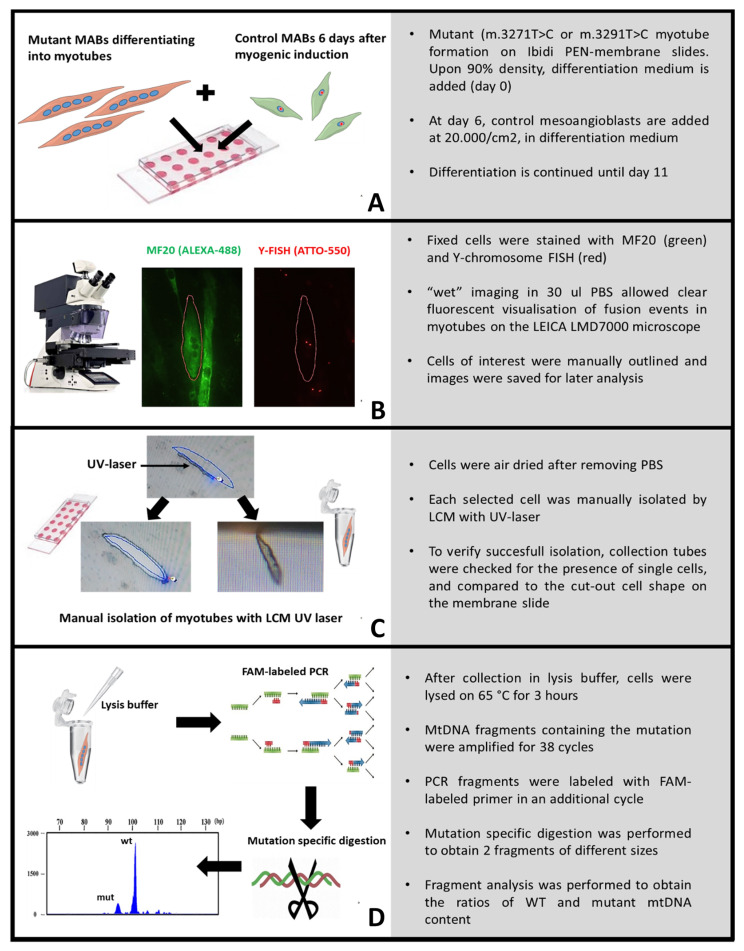
Schematic overview of fluorescent single-cell isolation and analysis. (**A**) mutant myotube culture and mixing of control MABs in Ibidi PEN-membrane slides for LCM, (**B**) representative images of selected cell-based multinucleated morphologies and subsequent selection on the presence of control nuclei (red Y-signal) in PBS, (**C**) laser capture process of selected cells after dehydration in brightfield mode, (**D**) cell manipulation protocol for subsequent mtDNA mutation load analysis.

To analyse if the fusion of wild-type MABs with mtDNA mutant myotubes resulted in the reduction of the mtDNA mutation load, we developed a procedure that enabled precise and efficient isolation of individual (fused) myotubes using laser capture microdissection (LCM) following MF20 and Y-chromosome fluorescent staining. Firstly, MUT1/MUT2 MABs were cultured until confluence on PEN-membrane slides, differentiation was induced by switching to differentiation medium (day 0), and wild-type MABs were added to the MUT1/MUT2 culture 6 days after induction of myogenic differentiation (Figure 4A). The MUT/wild-type mixed culture was then further differentiated until day 11 and subsequently fixated. After staining the Y-chromosome and myotube or cytosol staining using respectively MF20 or KDEL antibody, cells were visualized under the Laser Capture Microdissection (LCM) microscope with 30 µL PBS per well. Myotubes were selected based on the presence of ≥2 nuclei. Subsequently, the number of Y-chromosome positive and Y-chromosome negative nuclei per myotube was counted. The use of DAPI staining for nuclear visualization was excluded since the specialized PEN membrane designed for laser dissection displayed high levels of blue autofluorescence. Myotubes with both Y-positive and Y-negative nuclei were selected for LCM by manually outlining the cell edges, while the exact location was automatically saved by assigning each selected cell to a collector’s tube for subsequent dry laser cutting (Figure 4B). After selecting all myotubes of interest, images of both channels were made per myotube for later quantification. Next, the slide was dehydrated by removing the PBS and air-drying for 5 min. After each isolation step, the collection tube was checked for the presence of the dissected myotube and the resemblance to the original cell shape (Figure 4C). When present, the collection tubes containing 10 µL of cell lysis buffer were carefully closed, centrifuged at maximal speed, and lysed for 3 h. 

### 2.4. Fusion of Healthy MABs with m.3271T>C and m.3291T>C Myotubes Results in a Decreased mtDNA Mutation Load

**Figure 5:**
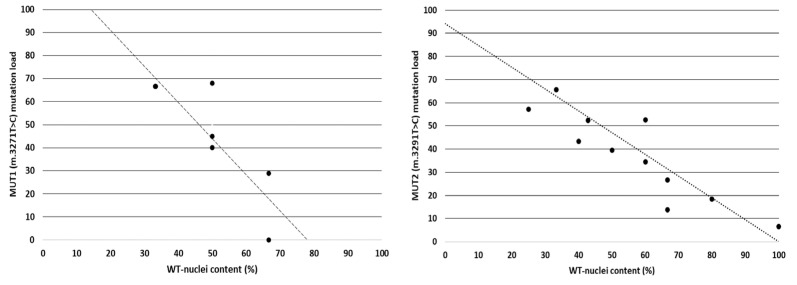
MUT1 (m.3271T>C) (*n* = 6) and MUT2 (m.3291T>C) (*n* = 11) mutation load (Y-axis) in individual fused myotubes relative to the fusion ratio with control MABs (X-axis). The black dotted line indicated the expected mutation load to fusion ratio based on the initial mtDNA mutation load in the parental MABs.

To assess whether the fusion of wild-type MABs with 94% m.3271T>C (MUT1) and 92% m.3291T>C (MUT2) mutant myotubes results in a reduced mtDNA mutation load relative to the fusion ratio, the mutation load per isolated myotube was assessed (Figure 4D). In total, the mutation load in isolated myotubes with different XX/XY fusion ratios was analysed. Figure 5 shows the m.3291T>C and m.3271T>C mtDNA mutation load in isolated myotubes. Both showed a reduction in mtDNA mutation load proportional to the wild-type-derived mtDNA content present in the fused myotube. 

## 3. Discussion

For heteroplasmic pathogenic mtDNA mutations, OXPHOS deficiency becomes clinically relevant when the mtDNA mutation load exceeds a (tissue)specific threshold, resulting in the manifestation of disease symptoms. Lowering the percentage of mutant mtDNA below this threshold by fusion of mtDNA-mutated myotubes with healthy mesoangioblasts might result in sufficient mitochondrial function within the cell or tissue to consolidate, revert or prevent symptoms [11,12]. This is the basis of our therapeutic strategy. In this study, we demonstrated, using a newly developed LCM procedure, that in vitro fusion of wild-type mesoangioblasts with mtDNA mutated (MUT) myotubes leads to a proportional reduction in mtDNA mutation load in individual fused myotubes.

Firstly, we demonstrated that wild-type-MABs were able to fuse with differentiating MUT-myotubes and fuse with undifferentiated MUT-MABs to form new hybrid wild-type-MUT myotubes, as well as myotubes consisting purely of wild-type nuclei, resembling the muscular environment. After studying the individual fusion ratios, we observed wild-type:MUT fusion ratios of 42.0 ± 17.1% (mean ± stdev) for the ratio of wild-type:MUT1 myotubes and 45.4 ± 23.4% (mean ± stdev) for the ratio of wild-type:MUT2 myotubes, which indicates that hybrid myotubes tend to fuse from equal amounts of wild-type and MUT cells. Moreover, the fusion between a wild-type MAB and a MUT-MAB occurred in 43.5% (wild-type-MUT1) and 42.3% (wild-type-MUT2) of all myotubes, indicating that fusion events occur relatively frequently between individual mesoangioblasts in an environment that favours myogenic differentiation. Previously, several studies focusing on the fusion ratios of human myoblasts with murine C2C12 cells pointed out that fusion after coculture occurs relatively frequently [13,14]. However, the use of a cytoplasmic labelling strategy to identify fusion events hampered a detailed insight into the actual fusion ratios within individual myotubes. By using an anti-human nuclear antibody staining, Sohn et al. demonstrated that fusion ratios in murine-human hybrid myotubes contained a comparable amount of both murine and human nuclei. Coculturing WT nascent murine myotubes (24 h on differentiation medium) with human myoblasts resulted in hybrid myotubes consisting of only 17.5% human nuclei, whereas coculturing WT murine myoblast with nascent human myotubes resulted in myotubes containing 44% human nuclei [15]. A study by Chen et al., in which human myoblasts and murine C2C12’s myoblasts were fused to study the contractile response of myotubes to electric pulse stimulation, found that approximately 15% of nuclei in the myotubes originated from human myoblasts [16]. As our results showed that for both mtDNA mutant myotubes cultures, fusion with wild-type mesoangioblasts resulted in average fusion ratios of almost 45%, these studies indicate that fusion ratios between myoblasts of different species can be variable, demonstrating the need for analysis at the level of the individual muscle fibres of a single species.

In order to assess the mtDNA mutation load in individual myotubes in vitro, we developed an LCM protocol in which both fluorescent imaging and single-cell isolation are combined. In contrast, most of the available LCM protocols are orientated at cell-cluster isolation from tissue sections [17,18,19,20,21]. When single myotube isolation is being performed, bright field microscopy is used, which hampers the selection for multiple fluorescently labelled probes, which we used to select for hybrid myotubes. Another limitation of previously published LCM protocols is the relatively low quality of fluorescent imaging, as LCM isolation requires dry rather than liquid or oil embed samples. These studies included fluorescent LCM imaging and used an overall cell or nucleus staining to select cells of interest instead of a more specific IHC staining [22,23]. One of our main adaptions to these LCM protocols was subsequently performing ‘wet’ and ‘dry’ imaging to improve image quality, as fluorescently imaging dry samples resulted in high background noise due to light scattering, hampering a trustworthy selection of cells of interest. Since the LCM microscope can save the position of marked regions, cells of interest can be traced back after air drying the sample slide. Another challenge that we encountered was the retention of the LCM sample in the membrane due to static electricity created by the laser. Therefore, we increased the humidity of the sample. We found that air drying the samples prior to LCM cutting, rather than the recommended ethanol dehydration, substantially increased the number of cells that were successfully collected into the collection tubes. Moreover, our LCM strategy could also be used following live-cell staining, e.g., analysis of mitochondrial membrane potential by TMRM staining.

After successfully isolating individual wild-type/MUT fused myotubes, we demonstrated that the m.3291T>C and m.3271T>C mutation load declined proportionally to the percentage of wild-type nuclei present in the myotube. This also implies that the amount of cytoplasm per nucleus and the number of mitochondria/mtDNA copies is comparable between the mesoangioblasts and myotubes. In line with our findings, Boulet et al. previously showed that coculturing only a small amount of healthy myoblasts with MERRF mutant myoblasts restored OXPHOS complex IV activity to near-normal levels [10]. For technical reasons, we were not able to perform comparable functional assays in the microdissected samples. But still, this data is promising in the context of the muscle stem cell therapy we are developing for carriers of mtDNA mutations using autologous, wild-type mesoangioblasts. The effectiveness of this novel stem cell therapy in improving cellular mitochondrial function in muscle in vivo largely depends on the mutation load, fusion rate, disease threshold, cytoplasmic fusion ratios, and the number of mitochondria in mesoangioblasts compared to the myotubes and myofibers. If the difference between the mutation load in muscle and the threshold of expression is large, this might require multiple fusion events in order to achieve a functionally relevant reduction. The same will apply if the muscle fibres have large numbers of nuclei and more mitochondria per unit cytoplasm than the wild-type mesoangioblasts. We are currently working on increasing the number of mitochondria in mesoangioblasts to improve the efficacy of our therapeutic approach. Our optimized protocol of combining individual myotube fusion assessment with LCM isolation for genetic analysis is of added value in predicting the effectiveness of future stem cell therapies and testing approaches to optimize this.

## 4. Materials and Methods

### 4.1. Cell Culture

Two MAB cell cultures with a high mtDNA mutation load were obtained from female carriers with the m.3271T>C mutation (MUT1) or m.3291T>C mutation (MUT2). As wild-type, MABs from a male m.3243A>G mutation carrier (M06) with <4% m.3243A>G mutation load and no detectable level of m.3271T>C or m.3291T>C were used. All MAB cultures were isolated from muscle biopsies and characterized as described [3]. All materials were obtained from Thermo Fisher Scientific unless stated otherwise. MABs were cultured in IMDM medium supplemented with 1× glutamine, 1× sodium-pyruvate, 1× insulin transferase selenium X, 1× non-essential amino acids, 0.2% 2-mercaptoethanol, 10% fetal bovine serum (Bodinco, Alkmaar, The Netherlands), 5 ng/mL FGF2 (Miltenyi Biotec, Leiden, The Netherlands) and 0.1% gentamycin, in a 37 °C humidified incubator in 4% O_2_ and 5% CO_2_ conditions.

### 4.2. Myogenic Differentiation and Fusion

The MABs were plated at 10,000 cells/cm^2^ onto hESC-Qualified Matrigel (Corning, Amsterdam, The Netherlands) (1:50) coated glass clover slips or Matrigel (1:50) coated 18-well polyethylene naphthalate (PEN) membrane slides (Ibidi, Gräfelfing, Germany) and grown until 95–100% confluent. Upon confluence, cells were washed with 1× PBS and incubated in a myogenic differentiation medium consisting of DMEM supplemented with 2% Horse serum and 0.1% gentamycin (day 0) and differentiated into myotubes until day 11. For fusion experiments, 20,000 cells/cm^2^ male wild-type M06 MABs in myogenic differentiation medium were added on day 6 to the differentiating myotubes in the 18-well PEN ibidi slides or on glass slides. On day 11, cultures were fixed in 3.7% paraformaldehyde, washed 3 times with 1× PBS for 5 min and kept in 1× PBS till analysis.

### 4.3. FISH and Immunohistochemistry

Fixed cell mixtures in 1xPBS were incubated in fresh 0.2% Triton-X for 30 min at room temperature and subsequently washed in 70%, 96%, and 99% ethanol. Once dehydrated, a Y-chromosome FISH probe was prepared as described previously [24] and was added to the Ibidi wells (100 μL probe/cm^2^), which were covered with small plastic cover slips or as a 10 μL droplet under the glass cover slips on a microscope slide. Samples were denatured on an 80 °C heating plate for 30 min and subsequently hybridized overnight at 37 °C in a humid chamber. After washing with 1x PBS, cells were blocked in 1% bovine serum albumin in 1× PBS for 1 h at room temperature and then incubated with 1:200 monoclonal MF20 (Myosin 4) antibody diluted in 0.2% Triton-X in PBS at 4 °C overnight. After three times washing with PBS-0.05% Triton-X, 1:1000 goat-anti-mouse 488 FITC (Abcam, Cambridge, UK) as a secondary antibody was incubated for 1 h at room temperature in 1×PBS-0.05%Tween, and subsequently washed 3 times in 1× PBS. Glass coverslips were mounted on microscope slides in glycerol DABCO with 1:1000 DAPI (Sigma-Aldrich, Amsterdam, Netherlands). Ibidi slides were kept in 1× PBS. The fusion ratio was analysed on a Leica SP5Confocal microscope with 63× magnification.

### 4.4. Laser Capture Microdissection

To collect individual myotubes, MF20-immunostained cells on ibidi slides we visualized under a fluorescent LEICA LMD6500 laser capture microdissection microscope with a green (488 nm) and red (555 nm) filter. Cells of interest were marked by tracing the outer edges of the cytosol manually, and coordinates and slide orientation were saved. For each cell, images of MF20 and Y-chromosome staining were saved for later analysis. Ibidi slides were dehydrated as described above and placed back in bright field mode. Cells were cut out using the following laser settings: laser power 10, aperture 12, and speed 8. Each cell was collected in a separate 200 μL tube containing 10 μL of lysis buffer solution (50 mM dithioethanol (Pharmacia Biotech, Uppsala, Sweden) and 200 mM NaOH (Sigma-Aldrich, Amsterdam, Netherlands) in 1× PBS).

### 4.5. mtDNA Mutation Load and Copy Number Assessment

Upon LCM, individual myotubes collected in lysis buffer were incubated for 3 h at 65 °C, then cooled down to 4 °C, after which 4 ul Tricine (20 mM pH 4.95 (Sigma-Aldrich, Amsterdam, Netherlands)) and 19.3 μL MiliQ were added. For the m.3291T>C mutation, PCR-I was performed in 50 μL reaction volume containing 1× PCR buffer, 1.5 mM MgCl_2_, 1U Taq DNA polymerase (GC Biotech Bioline, Waddinxveen, Netherlands), 0.1 mM dNTPs (Pharmacia), 0.3 μM reverse primer and 0.06 μM forward primer (m.3291T>C forward: 5′ CAACTTAGTATTATACCCACAC; m.3291T>C reverse: 5′ TTTCGTTCGGTAAGCATTAG), and 3 μL of cell lysis product. PCR was performed on a Biometra T1 professional thermocycler (Westburg, Leusden, The Netherlands) using the following program: 5 min at 94 °C; 38 cycles of 1 min at 92 °C, 45 s at 60 °C, 45 s at 72 °C; 7 min at 72 °C. Following PCR-I, 15 μL of PCR-I product was mixed with 0.3 μM FAM-labelled forward primer (FAM-caacttagtattatacccacac), 1× PCR buffer, 1.5 mM MgCl_2_, 1U Taq DNA polymerase (GC Biotech Bioline, Waddinxveen, The Netherlands), 0.05 mM dNTPs (Pharmacia Biotech, Uppsala, Sweden) in a 50 μL reaction, and amplified for one cycle using the following program: 5 min at 94 °C; 1 min at 92 °C, 45 s at 60 °C, 45 s at 72 °C; 7 min at 72 °C. Digestion of PCR-II product was performed in a 50 μL reaction containing 10U enzyme (For m.3271T>C: NEB AflII, 5′…C-TTAAG…3′, 3′…GAATT-C…5′; for m.3291T>C: NEB MluCI, 5′…-AATT…3′, 3′…TTAA-…5′), 20 μL PCR-II product and 1× NEB cutsmart enzyme according to manufacturer’s protocol, and the digested product was purified with the innoPREP PCR pure kit (Analytik-Jena, Jena, Germany). The mtDNA mutation load was assessed on an ABI Prism 3730 Genetic Analyser, and data were processed with Gene-scan Analysis 3.7 and Genemarker analysis v.2.6.3 software. The m.3271T>C mutation load was assessed by real-time quantification of the number of mutated and wild-type mtDNA copies per sample using a mutation-specific and wild-type-specific forward primer. Per reaction, 2.5 μL of four-times diluted cell lysate was mixed with 7.5 μL containing 1× Sensimix Sybr Hi-Rox reagent (Bioline, Waddinxveen, Netherlands) and 375 nM forward (m.3271T>C mutant: GTAATCGCATAAAACTTAAAACC, m.3271T>C wild-type: GTAATCGCATAAAACTTAAAACT) and reverse primer (TTTCGTTCGGTAAGCATTAG). Amplification was performed on the Roche Light cycler 480 using the following settings: 10 min 95 °C, 40 cycles of 15 s 95 °C and 1 min 60 °C. The mtDNA copy number was assessed by comparing the ratio of mtDNA (D-loop primers; forward 5′ CATCTGGTTCCTACTTCAGGG, reverse 5′ TGAGTGGTTAATAGGGTGATAGA) to n-DNA (B2M primers; forward 5′ TGCTGTCTCCATGTTTGATGTATCT, reverse 5′ TGCTGTCTCCATGTTTGATGTATCT) in a real-time quantitative amplification reaction on a Roche LightCycler 480. Each reaction contained 5 ng DNA, 1x Sensimix Sybr Hi-ROX (Bioline, Waddinxveen, Netherlands) and 375 nM of forward and reverse primer and was amplified using the following program: 95 °C for 10 min, 40× 95 °C for 15 s and 60 °C for 1 min.

## Figures and Tables

**Table 1:** Distribution of nuclei in subpopulations at myogenic differentiation day 11 after mixing wild-type MABs with mutant myotubes.

% of Population Present	MUT1 + Wild-Type MABs	MUT2 + Wild-Type MABs
**Total MABs content**	**71.0%**	**46.0%**
mutant MABs content (1 Y-neg. nucleus)	61.5%	32.2%
wild-type MABs content (1 Y-pos. nucleus)	9.5%	13.8%
**Total myotube content**	**29.8%**	**54.0%**
mutant myotubes (only ≥2 Y-neg. nuclei in MF20+ myotube)	17.9%	17.2%
wild-type myotubes (only ≥2 Y-pos. nuclei in MF20+ myotube)	1.1%	0.9%
Fused myotubes (≥1 Y-pos. and ≥2 Y-neg. nuclei) (% wild-type nuclei/% mutant nuclei)	8.0% *(2.0%/6.1%)*	32.5% *(9.7%/22.8%)*
MABs-fusion (≥1 Y-pos. and 1 Y-neg. nucleus) (% wild-type nuclei/% mutant nuclei)	2.8% *(1.1%/1.7%)*	3.4% *(2.3%/1.1%)*
**Total % mutant (Y-neg) nuclei in population**	**86.3%**	**73.3%**
**Total % wild-type (Y-pos.) nuclei in population**	**13.7%**	**26.7%**

*n* = 21 images for MUT1 (m.3272T>C), *n* = 34 images for MUT2 (m.3291T>C), Y-neg. is Y-chromosome-negative nucleus, Y-pos. is Y-chromosome-pos. nucleus.

## Data Availability

All data generated and/or analyzed during this study are included in this manuscript.

## References

[1] Gorman G.S., Schaefer A.M., Ng Y., Gomez N., Blakely E.L., Alston C.L., Feeney C., Horvath R., Yu-Wai-Man P., Chinnery P.F. (2015). Prevalence of nuclear and mitochondrial DNA mutations related to adult mitochondrial disease. Ann. Neurol..

[2] Wai T., Ao A., Zhang X., Cyr D., Dufort D., Shoubridge E.A. (2010). The role of mitochondrial DNA copy number in mammalian fertility. Biol. Reprod..

[3] van Tienen F., Zelissen R., Timmer E., van Gisbergen M., Lindsey P., Quattrocelli M., Sampaolesi M., Mulder-den Hartog E., de Coo I., Smeets H. (2019). Healthy, mtDNA-mutation free mesoangioblasts from mtDNA patients qualify for autologous therapy. Stem Cell Res. Ther..

[4] Fu K., Hartlen R., Johns T., Genge A., Karpati G., Shoubridge E.A. (1996). A novel heteroplasmic tRNAleu(CUN) mtDNA point mutation in a sporadic patient with mitochondrial encephalomyopathy segregates rapidly in skeletal muscle and suggests an approach to therapy. Hum. Mol. Genet..

[5] Spendiff S., Reza M., Murphy J.L., Gorman G., Blakely E.L., Taylor R.W., Horvath R., Campbell G., Newman J., Lochmüller H. (2013). Mitochondrial DNA deletions in muscle satellite cells: Implications for therapies. Hum. Mol. Genet..

[6] Shoubridge E.A., Johns T., Karpati G. (1997). Complete Restoration of a Wild-Type mtDNA Genotype in Regenerating Muscle Fibres in a Patient with a tRNA Point Mutation and Mitochondrial Encephalomyopathy. Hum. Mol. Genet..

[7] Sampaolesi M., Blot S., D’Antona G., Granger N., Tonlorenzi R., Innocenzi A., Mognol P., Thibaud J.L., Galvez B.G., Barthélémy I. (2006). Mesoangioblast stem cells ameliorate muscle function in dystrophic dogs. Nature.

[8] Cossu G., Previtali S.C., Napolitano S., Cicalese M.P., Tedesco F.S., Nicastro F., Noviello M., Roostalu U., Natali Sora M.G., Scarlato M. (2015). Intra-arterial transplantation of HLA-matched donor mesoangioblasts in Duchenne muscular dystrophy. EMBO Mol. Med..

[9] So K.H., Han Y.J., Park H.Y., Kim J.G., Sung D.J., Bae Y.M., Yang B.C., Park S.B., Chang S.K., Kim E.Y. (2011). Generation of functional cardiomyocytes from mouse induced pluripotent stem cells. Int. J. Cardiol..

[10] Boulet L., Karpati G., Shoubridge E.A. (1992). Distribution and threshold expression of the tRNA(Lys) mutation in skeletal muscle of patients with myoclonic epilepsy and ragged-red fibers (MERRF). Am. J. Hum. Genet..

[11] Rossignol R., Faustin B., Rocher C., Malgat M., Mazat J.P., Letellier T. (2003). Mitochondrial threshold effects. Biochem. J..

[12] Rai P.K., Craven L., Hoogewijs K., Russell O.M., Lightowlers R.N. (2018). Advances in methods for reducing mitochondrial DNA disease by replacing or manipulating the mitochondrial genome. Essays Biochem..

[13] Dellavalle A., Maroli G., Covarello D., Azzoni E., Innocenzi A., Perani L., Antonini S., Sambasivan R., Brunelli S., Tajbakhsh S. (2011). Pericytes resident in postnatal skeletal muscle differentiate into muscle fibres and generate satellite cells. Nat. Commun..

[14] Berry S.E., Liu J., Chaney E.J., Kaufman S.J. (2007). Multipotential mesoangioblast stem cell therapy in the *mdx/utrn*^−/−^ mouse model for Duchenne muscular dystrophy. Regen. Med..

[15] Sohn R.L., Huang P., Kawahara G., Mitchell M., Guyon J., Kalluri R., Kunkel L.M., Gussoni E. (2009). A role for nephrin, a renal protein, in vertebrate skeletal muscle cell fusion. Proc. Natl. Acad. Sci. USA.

[16] Chen W., Nyasha M.R., Koide M., Tsuchiya M., Suzuki N., Hagiwara Y., Aoki M., Kanzaki M. (2019). In vitro exercise model using contractile human and mouse hybrid myotubes. Sci. Rep..

[17] Amini P., Ettlin J., Opitz L., Clementi E., Malbon A., Markkanen E. (2017). An optimised protocol for isolation of RNA from small sections of laser-capture microdissected FFPE tissue amenable for next-generation sequencing. BMC Mol. Biol..

[18] Gautam V., Singh A., Singh S., Sarkar A.K. (2016). An Efficient LCM-Based Method for Tissue Specific Expression Analysis of Genes and miRNAs. Sci. Rep..

[19] Tayade C., Edwards A.K., Bidarimath M., Croy B.A., Yamada A.T., DeMayo F.J., Adamson S.L. (2014). 48—Laser Capture Microdissection. The Guide to Investigation of Mouse Pregnancy.

[20] Murakami H., Liotta L., Star R.A. (2000). IF-LCM: Laser capture microdissection of immunofluorescently defined cells for mRNA analysis rapid communication. Kidney Int..

[21] Shi X., Kleeff J., Zhu Z.W., Schmied B., Tang W.H., Zimmermann A., Buchler M.W., Friess H. (2003). Gene-expression analysis of single cells-nested polymerase chain reaction after laser microdissection. World J. Gastroenterol..

[22] Khodosevich K., Inta D., Seeburg P.H., Monyer H. (2007). Gene expression analysis of in vivo fluorescent cells. PLoS ONE.

[23] Williams D.L., Schwartz M.W., Bastian L.S., Blevins J.E., Baskin D.G. (2008). Immunocytochemistry and laser capture microdissection for real-time quantitative PCR identify hindbrain neurons activated by interaction between leptin and cholecystokinin. J. Histochem. Cytochem..

[24] Schouten H.C., Hopman A.H., Haesevoets A.M., Arends J.W. (1995). Large-cell anaplastic non-Hodgkin’s lymphoma originating in donor cells after allogenic bone marrow transplantation. Br. J. Haematol..

